# Regulation of Cell Proliferation and Migration in Glioblastoma: New Therapeutic Approach

**DOI:** 10.3389/fonc.2013.00053

**Published:** 2013-03-18

**Authors:** Yangjin Kim

**Affiliations:** ^1^Department of Mathematics, Konkuk UniversitySeoul, South Korea

**Keywords:** glioblastoma, cell migration and proliferation, miR-451, AMPK, cancer invasion and therapy

## Abstract

Glioblastoma is the most aggressive brain cancer with the poor survival rate. A microRNA, miR-451, and its downstream molecules, CAB39/LKB1/STRAD/AMPK, are known to play a critical role in regulating a biochemical balance between rapid proliferation and invasion in the presence of metabolic stress in microenvironment. We develop a novel multi-scale mathematical model where cell migration and proliferation are controlled through a core intracellular control system (miR-451-AMPK complex) in response to glucose availability and physical constraints in the microenvironment. Tumor cells are modeled individually and proliferation and migration of those cells are regulated by the intracellular dynamics and reaction-diffusion equations of concentrations of glucose, chemoattractant, extracellular matrix, and MMPs. The model predicts that invasion patterns and rapid growth of tumor cells after conventional surgery depend on biophysical properties of cells, dynamics of the core control system, and microenvironment as well as glucose injection methods. We developed a new type of therapeutic approach: effective injection of chemoattractant to bring invasive cells back to the surgical site after initial surgery, followed by glucose injection at the same location. The model suggests that a good combination of chemoattractant and glucose injection at appropriate time frames may lead to an effective therapeutic strategy of eradicating tumor cells.

## 1 Introduction

Glioblastoma multiforme (GBM) is the most common and aggressive form of primary brain tumor with the median survival time of approximately 1 year from the time of diagnosis (Demuth and Berens, 2004; Stylli et al., 2005; Jacobs et al., 2011). GBMs are characterized by rapid proliferation and aggressive invasion into surrounding normal brain tissue, which leads to inevitable recurrence after surgical resection of the primary tumor (Chintala et al., 1999). Surgery is the primary treatment method, generally followed by inefficient radiotherapy and chemotherapy. Innovative therapeutic approaches of targeting these invasive cells are needed in order to improve clinical outcome (Davis and McCarthy, 2001). Glioblastoma cells are encountered with many challenges such as hypoxia (lack of oxygen), acidity, and limited nutrient availability as tumor growth is proceeded. To keep up with rapid growth, tumor cells need to adapt to these biochemical changes in the harsh microenvironment (Godlewski et al., 2010a). In order to overcome these challenges and sustain their rapid growth, cancerous cells change their typical metabolism (oxidative phosphorylation and anaerobic glycolysis) to inefficient metabolic machinery [high levels of glucose uptake and lactate production; *Warburg Effect* (Warburg, 1956; Kim and Dang, 2006)].

**Figure 1:**
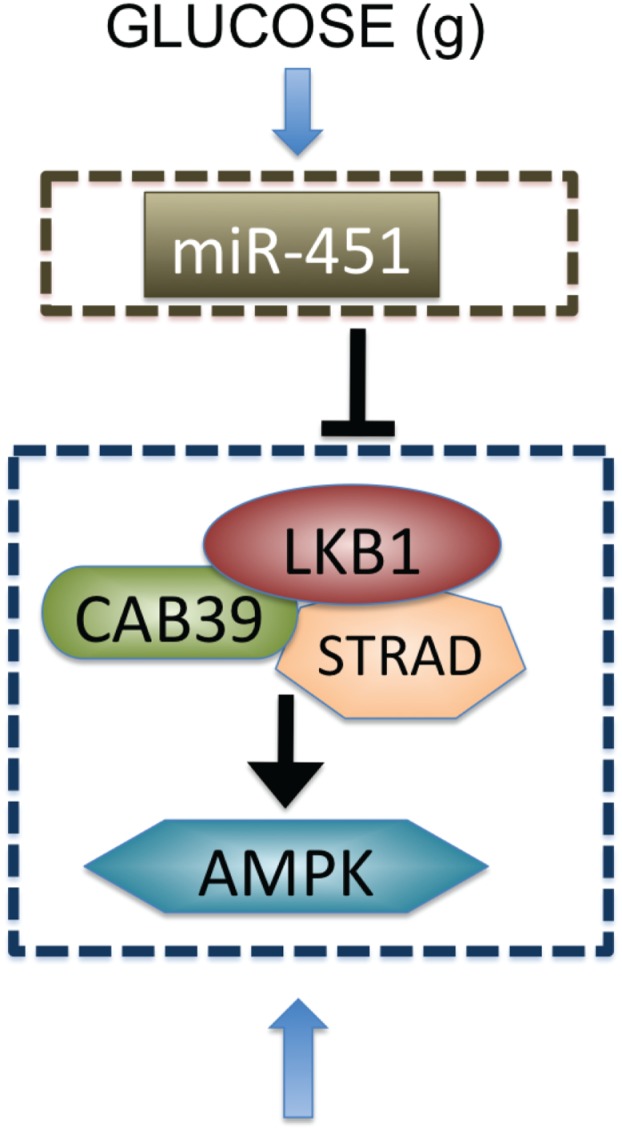
**Biological observation for regulation of miR-451-AMPK complex (Godlewski et al., 2010a)**.

The Krebs, or tricarboxylic acid (TCA) cycle is a main step for generating an energy source, ATP, in non-hypoxic normal cells. While this effective way of metabolism is used by differentiated cells, tumor cells favor a seemingly less effective way of metabolism, aerobic glycolysis (Heiden et al., 2009) due to production of lactic acid, and consumption of large amounts of glucose (Kim and Dang, 2006). Adapting this aerobic glycolysis (Gatenby and Gillies, 2004), cancer cells appear to have an advantage of not having to rely on oxygen for energy source in hypoxic (hostile) microenvironment (Gatenby and Gillies, 2004; Kim and Dang, 2006). Better understanding of basic mechanism of glycolysis and intracellular dynamics may provide better clinical outcomes. For example, inhibition of glycolysis may prevent drug resistance (Xu et al., 2005). Cancer cells also adapt angiogenesis and migration as a way of ensuring an adequate glucose supply (Godlewski et al., 2010a). However, appropriate intracellular responses to glucose withdrawal are a crucial component of adaptation in order to survive periods of metabolic stress and maintain viability as a tumor grows (Jones and Thompson, 2009). The 5′-adenosine monophosphate activated protein kinase (AMPK) pathway is the major cellular sensor of energy availability (Hardie, 2007) and is activated in the presence of metabolic stress as a way of promoting glucose uptake and energy conservation (Hardie, 2007). Dysregulation of miRNAs, 22 nucleotide single-stranded non-coding RNAs (Bartel, 2009), has been associated with oncogenic activities and tumor suppressor (Esquela-Kerscher and Slack, 2006) in many cancer types, including glioblastoma where alterations in miRNA expression induces tumorigenesis (Godlewski et al., 2008; Lawler and Chiocca, 2009). For example, miR-21 promote glioma invasion by down-regulation of inhibitors of matrix metalloprotease (MMP) (Gabriely et al., 2008). In a recent paper, Godlewski et al. (2010a) found that a particular microRNA, miR-451, determines glioma cell motility and proliferation by regulating its counterpart, AMPK signaling component (CAB39/LKB1/AMPK), in response to various glucose levels. While normal glucose led to up-regulation of miR-451 expression and rapid cell proliferation, deprived glucose induced down-regulation of miR-451 and elevated cell migration. Godlewski et al. (2010a) also found mutual antagonism between miR-451 activity and AMPK complex levels, which was modeled using a mathematical model in Kim et al. (2011a). See Figure 1.

Invasion of glioma cells leads to treatment failure due to poor screening of invasive individual cells by the standard clinical device and difficulty in complete elimination of the migratory cells in typical brain surgery, causing tumor recurrence (Chintala et al., 1999). Many factors may contribute to glioma cell motility in the brain tissue. Extra cellular matrix (ECM) may stimulate glioma invasion in a process known as haptotaxis. Haptotactic process is suggested to be activated by pre-existing brain components and remodeling of the ECM via proteolysis (Chintala et al., 1999; Jaalinoja et al., 2000; Choe et al., 2002). Glioma cell’s motility is also influenced by various chemoattractants, which include ligands of scatter factor/hepatocyte growth factor (SF/HGF) (Lamszus et al., 1998), the EGF family (Lund-Johansen et al., 1990), the TGF-*β* family (Platten et al., 2001), SDF-1 (Zhou et al., 2002), and certain lipids (Young and Brocklyn, 2007). We note that other authors studied the action of HGF or scatter factor on cell migration (Tamagnone and Comoglio, 1997; Luca et al., 1999; Stella and Comoglio, 1999; Trusolino and Comoglio, 2002; Scianna et al., 2009). Beside these factors, other cell types such as microglia can also provide indirect stimulation of cell migration by secreting matrix components and chemoattractants (Watters et al., 2005). Glioma cell migration may be regulated by specific substrates and structures in the brain as well. For instance, glioma cells are also known to follow preferred dispersion paths such as white matter tracts or the basal lamina of blood vessels. Invasion patterns of glioma cells in three-dimensional tumor spheroids were studied in Kim et al. (2009) where the migration patterns exhibit a gradual shift from branching to dispersion and depend on three key parameters (cell–cell adhesion strength, haptotactic parameter, and chemotactic sensitivity). There are several publications based on a diffusion model (Swanson et al., 2003; Harpold et al., 2007).

Other authors investigated the transition between migration and proliferation using kinetic or diffusion models (Chauviere et al., 2010; Hatzikirou et al., 2010; Pham et al., 2012). A general review on hybrid models of tumor growth can be found in Rejniak and Anderson (2011). In the present paper, the detailed dynamics of a core control system (miR-451-AMPK) at each cell site is embedded in a hybrid model and is linked to extracellular glucose molecules which diffuse to brain tissue. In the hybrid model, tumor cells either migrate or proliferate in response to biochemical signals such as glucose and chemoattractants. Migratory cells are attracted to chemotactic source and secrete MMPs to degrade extracellular matrix (ECM). We show how the spatial migrating patterns of glioma cells can be controlled by the core system in the absence and presence of chemotactic source after initial surgery and explore how injection of glucose and chemoattractants could be manipulated for better therapeutic options. More importantly, we use the current model to test hypotheses on chemotaxis-glucose-driven therapy, i.e., eradicating “invisible” invasive cells after surgery. We propose that injection of chemoattractants after surgery followed by glucose injection at the tumor site would bring migratory glioma cells back to the surgical site and make them detectable by MRI, and the follow-up surgery may improve clinical outcomes by eradicating the remaining growing tumor cells.

In Section 2 we introduce a multi-scale mathematical model. In Section 3, we present the results from the hybrid model. Discussion and future work are provided in Section 4. Parameter estimation and non-dimensionalization of the model are given in Appendix.

## 2 Materials and Methods

**Figure 2:**
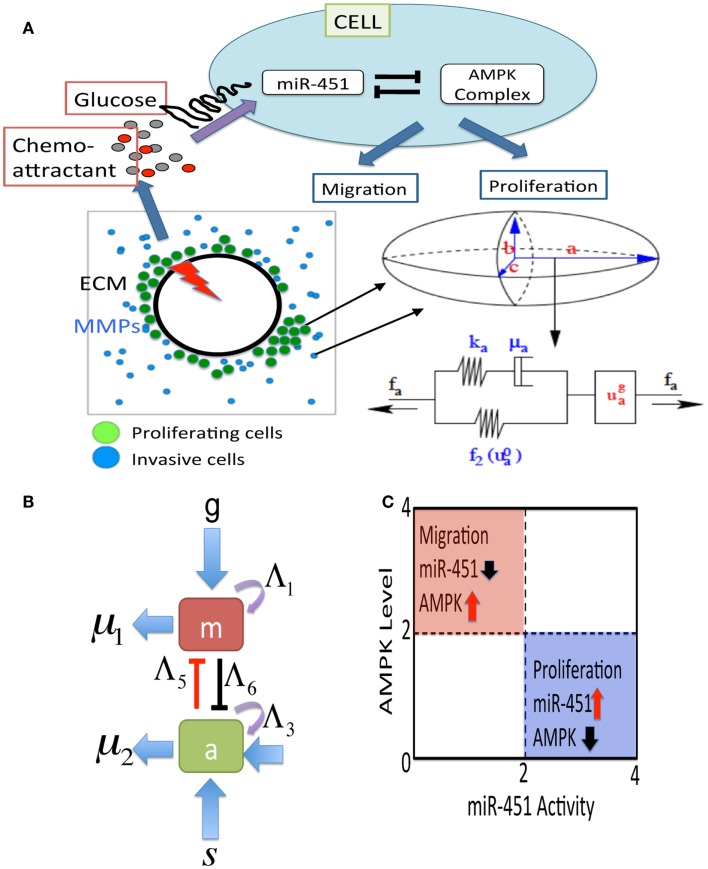
**(A)** A schematic of the hybrid model for therapeutic approaches. (Top) Simplified model of the core control system (miR-451 and AMPK complex) in response to various glucose levels at a tumor cell site. The core system determines the cell fate, either proliferation or migration. Chemoattractants in the brain tissue determine the migration direction of the cell. (Bottom, Left) Model domain: some migratory cancer cells (blue) near the tumor core site are activated to become a proliferative one (green) via miR-451-AMPK regulation in response to glucose injection levels (red thunder) at the center of the tumor site. (Bottom, right) changes in the length of the *a*-axis of a cell (the ellipsoid) under a given force (*f_a_*; arrow) consist of the passive change in the first component (a Maxwell element in parallel with a non-linear spring) and the change due to the growth ($u_{a}^{g}$). The growth component depends on the levels of miR-451 and AMPK complex, and the force (*f_a_*). The mechanical and growth elements are the same along all axes. **(B)** miR-451 activity and levels of its target complex (CAB39/LKB1/AMPK) were represented by “*m*” and “*a*,” respectively. **(C)** The migratory and proliferative regions based on miR-451 activity and AMPK levels.

In this section, we introduce a multi-scale mathematical model of regulation of cell proliferation and migration in glioblastoma. We consider a brain tissue, Ω = [0, *L*]^2^, with glioblastoma tumor initially occupying a sphere $\Omega_{c}^{0}=\left\{x\text{: —}x\text{—}<R_{0},R_{0}<L\right\}$, where *R*_0_ is the initial radius of the tumor spheroid. A schematic of the hybrid model is shown in Figure 2. We first introduce the cell-mechanics part of the model.

### 2.1 The cell-mechanics

The mechanical behavior of individual cells is based on the models developed by Dallon and Othmer (2004) and Kim et al. (2007, 2011b). The forces on a cell in the model include (i) the dynamic drag forces from adhesive bonds with neighboring cells, (ii) the active forces **T**_*i*_ exerted on the substrate or neighboring cells and the reaction force (**M**_*j,i*_), (iii) static friction force **S**_*j,i*_ for rigid attachment between cells or between a cell and the substrate. (See DO for a more detailed discussion of all forces involved.) The total force on the *i*th cell is then given by

(1) $$F_{i}=\underset{j\in {𝒩}_{i}^{a}}{\sum }M_{j,i}+\underset{j\in {𝒩}_{i}^{a}}{\sum }T_{i}+\underset{j\in {𝒩}_{i}^{d}}{\sum }\mu_{ij}\left(v_{j}-v_{i}\right)+\underset{j\in {𝒩}_{i}^{s}}{\sum }S_{j,i}$$

where ${𝒩}_{i}^{a}$ denotes the neighbors of *i*, including the substrate, upon which it can exert traction, ${𝒩}_{i}^{d}$ is the set of “cells” (which includes substrate and extracellular matrix) that interact with *i* via a frictional force, and ${𝒩}_{i}^{s}$ denotes the set of cells that statically bind to cell *i*. These force balance equations allow us to calculate all forces involved and track down locations of all cells in addition to biophysical response of the cells.

There are two different kinds of cells involved-proliferative one and motile one. The basic mechanical scheme of cell proliferation is modeled as in Kim et al. (2007) and the basic algorithm for motile cells is introduced as in Dallon and Othmer (2004). The cells are treated as oriented ellipsoids and cytoplasm is considered as an incompressible, viscoelastic solid. When growth is off, their volume is constant under all deformations. However, growth component ($u_{g}^{a}$) is included in series the active response and the passive forces. (See Figure 2A.) We use the multiplicative form of the growth rate function for the *i*-th axis given by

$${\left(u_{i}^{g}\right)}^{\prime }=f\left(\sigma \right)P\left(M,A\right)$$

where *σ* is the force acting on the cell and *P* is a function of the miR-451 activity (*M*) and AMPK levels (*A*). The growth function *f* (*σ*) is defined so that cells can grow under sufficiently small tensile and compressive forces (Kim et al., 2007, 2011b). The relationship between growth and stresses are complex and further detailed modeling work is needed. Cell proliferation may depend on up- or down-regulation of intracellular players that control the cell cycle. In the present work, we assume that the core system determines cell proliferation, i.e., a cell proliferates when miR-451 (AMPK) is up-regulated (down-regulated) at the cell site. Then, the function *P*(*M*, *A*) is defined as

(2) $$P\left(M,A\right)=\left\{\begin{array}{cc} 1\; & \text{if }M>th_{M},A<th_{A}\\ 0\; & \text{otherwise}\\ \; \end{array}\right.$$

where *th_M_*, *th_A_* are threshold values of the miR-451 and AMPK levels that will be introduced in Section 2.3. The active force **T**_*i*_ of cell *i* is given by

(3) $$T_{i}=\phi \left(M_{i}\right)\frac{\nabla C}{\sqrt{K_{C}+|\nabla C{|}^{2}}}$$

where *C* is the concentration of a chemoattractant. Here, the indicator function *ϕ*(*M*) is given by

(4) $$\phi \left(M\right)=\left\{\begin{array}{cc} r_{n}F_{0}\; & \text{ if }M<th_{M},A>th_{A},\text{ cell without}\\ \; & \;\text{physical constraints,}\\ 0\; & \text{otherwise},\\ \; \end{array}\right.$$

where *F*_0_ is the basal magnitude of the active force (0 ≤ |**T**_*i*_| ≤ *F*_0_) and *r_n_* is a random number in [0.8, 1.2]. Therefore, the active force is completely turned off for proliferative cells (*M_i_* > *th_M_*, *A* < *th_A_*), cells under physical constraints (a cell completely surrounded by neighboring cells), or in the absence of chemotactic signal (∆*C* = 0).

### 2.2 Reaction-diffusion

We let *G*(**x**, *t*), *C*(**x**, *t*), ρ(**x**, *t*), *P*(**x**, *t*) be the concentrations of glucose, a chemoattractant of glioma cells, ECM, and MMPs, respectively, at space **x** and time *t*. Governing equations of all variables are given by

(5) $$\begin{array}{llll} \frac{\partial G}{\partial t} & =\underset{\text{Diffusion}}{\underset{︸}{D_{G}\Delta G}}+\underset{\text{Injection}}{\underset{︸}{\overset{N_{G}}{\underset{j=1}{\sum }}\lambda_{in}^{G}I_{\left[t_{j}^{G},t_{j}^{G}+\tau_{d}^{G}\right]\times \Omega_{\epsilon }}}} & & \\ & \;+\underset{\text{Input}}{\underset{︸}{\lambda_{b}\eta_{1}\left(x,G\right)}}-\underset{\text{Consumption}}{\underset{︸}{\lambda_{c}\eta_{2}\left(x,G\right)}}-\underset{\text{Removal}}{\underset{︸}{\mu_{G}G}}\text{ in }\Omega , \end{array}$$

(6) $$\begin{array}{ll} \frac{\partial C}{\partial t} & =\underset{\text{Diffusion}}{\underset{︸}{D_{C}\Delta C}}+\underset{\text{Injection}}{\underset{︸}{\overset{N_{C}}{\underset{j=1}{\sum }}\lambda_{in}^{C}I_{\left[t_{j}^{C},t_{j}^{C}+\tau_{d}^{C}\right]\times \Omega_{\epsilon }}}}-\underset{\text{Decay}}{\underset{︸}{\mu_{C}C}}\text{ in }\Omega , \end{array}$$

(7) $$\begin{array}{ll} \frac{\partial \rho }{\partial t} & =-\underset{\text{Degradation}}{\underset{︸}{\lambda_{1}P\rho }}+\underset{\text{Release/reconstruction}}{\underset{︸}{\lambda_{2}\rho \left(1-\frac{\rho }{\rho_{*}}\right)}}\text{ in }\Omega , \end{array}$$

(8) $$\begin{array}{ll} \frac{\partial P}{\partial t} & =\underset{\text{Diffusion}}{\underset{︸}{D_{P}\Delta P}}+\underset{\text{Production by cells}}{\underset{︸}{\lambda_{3}\eta_{3}\left(x,P\right)}}-\underset{\text{Decay}}{\underset{︸}{\mu_{P}P}}\text{ in }\Omega , \end{array}$$

where *D_G_*, *D_C_*, *D_P_* are the diffusion coefficients of glucose, chemoattractant, and MMPs, respectively, $\lambda_{in}^{G}$ ($\lambda_{in}^{G}$) is the glucose (chemoattractant) injection rate on a subdomain Ω{_ϵ_ over time intervals [$t_{j}^{G},\;t_{j}^{G}+\;\tau_{d}^{G}$], *j* = 1, …, *N_G_* ([$t_{j}^{C},\;t_{j}^{C}+\;\tau_{d}^{C}$], *j* = 1, …, *N_C_*) with a period *τ^G^* (*τ^C^*) and duration $\tau_{d}^{G}$ ($\tau_{d}^{C}$) after the initial surgery at *t* = *t_S_* ($t_{1}^{G}>t_{S}$), *λ_b_* is the glucose flux from a blood flow, *λ_c_* is the consumption rate of glucose by tumor cells, *λ*_1_ is the degradation rate of ECM by MMPs, *λ*_2_ is the release/reconstruction rate of ECM, *λ*_3_ is the secretion rate of MMPs by tumor cells, *μ_G_* is the glucose removal rate from the system via blood flow and glucose consumption in the surrounding tissue (Chiro et al., 1982; Rozental et al., 1991; Goldman et al., 1996; Aronen et al., 2000; Valle-Casuso et al., 2012), *μ_C_*, *μ_P_* are decay rates of chemoattractant and MMPs, respectively. Here, indicator functions (η_1_, η_2_, η_3_) are given by

$$\begin{array}{llll} \eta_{1}\left(x,G\right) & =\left\{\begin{array}{cc} 1\; & \text{blood vessel}\\ 0\; & \text{otherwise}\\ \; \end{array}\right.,\;\eta_{2}\left(x,G\right)=\left\{\begin{array}{cc} 1\; & \text{tumor}\\ 0\; & \text{otherwise}\\ \; \end{array}\right., & & \\ \eta_{3}\left(x,P\right) & =\left\{\begin{array}{cc} 1\; & \text{invasive cells}\\ 0\; & \text{otherwise}.\\ \; \end{array}\right. & & \end{array}$$

We also assume no flux (Neumann) boundary conditions $\frac{\partial G}{\partial \nu }\;=\;0,\frac{\partial C}{\partial \nu }=0,\frac{\partial P}{\partial \nu }=0$, on ∂ Ω. The reaction-diffusion equations (5–8) are solved on the regular grid using the alternating-direction implicit (ADI) method and the non-linear solver *nksol* for algebraic systems. A typical spatial grid size used is *h_x_* = *h_y_* = 0.01 on a square domain [0, 1] × [0, 1]. An adaptive time stepping method is used. Table 1 lists parameter values and references values for the equations (5)-(8).

**Table 1:** **Values of reference variables and parameters used in the hybrid model**.

Var	Description	Value	Refs.
**DIFFUSION COEFFICIENTS**			
*D_G_*	Glucose	6.7 × 10^−7^cm^2^/s	Jain (1987)
*D_C_*	Chemoattractant (EGF)	1.66 × 10^−6^ cm^2^/s	Thorne et al. (2004)
*D_P_*	MMPs	8.0 × 10^−9^ cm*^2^*/s	Saffarian et al. (2004)
**PRODUCTION/DECAY/CONSUMPTION RATES**			
*λ*_2_	ECM reconstruction/remodeling rate	5.6 × 10^−3^ s^−1^	TW
*λ*_3_	MMP production rate	5.8	
*λ_c_*	Glucose consumption rate by tumor	0.8 pg/cell/min	TW
*μ_G_*	Removal rate of glucose in brain tissue	0.0034 min^−1^	TW
*μ_C_*	Decay rate of chemoattractant (EGF)	8.02 × 10^−6^ s^−1^	Kudlow et al. (1986)
*λ*_1_	ECM degradation rate by MMPs	3.0 × 10^4^ cm^3^ g^−1^ s^−1^	TW
*μ_P_*	Decay rate of MMPs	5.0 × 10^−5^ s^−1^	TW
**REFERENCE VALUES**			
*T*	Time	1 h	
*L*	Length	2.0 mm	
G*	Glucose concentration	4.5 × 10^−3^ g/cm^3^	Deisboeck et al. (2001), Sander and Deisboeck (2002), Godlewski et al. (2010a)
C*	Chemoattractant (EGF) concentration	1.0 × 10^−8^ g/cm^3^	Boccardo et al. (2003), Sadlonova et al. (2005)
ρ*	ECM concentration	1.0 × 10^−3^ g/cm^3^	Kaufman et al. (2005), Stein et al. (2007)
P*	MMP concentration	1.0 × 10^−7^ g/cm^3^	Annabi et al. (2005)

### 2.3 Mathematical modeling of miR-451-AMPK control

The core control model of miR-451 activity and AMPK levels introduced in Kim et al. (2011a) was integrated into the hybrid model. Based on biological observations, we write the phenomenological equations for the rate change of those key molecules (*m*, *a*) as follows:

(9) $$\begin{array}{ll} \frac{dm}{dt} & =\lambda_{g}g+\frac{\Lambda_{1}\Lambda_{2}^{2}}{\Lambda_{2}^{2}+\Lambda_{5}a^{2}}-\mu_{1}m, \end{array}$$

(10) $$\begin{array}{ll} \frac{da}{dt} & =s+\frac{\Lambda_{3}\Lambda_{4}^{2}}{\Lambda_{4}^{2}+\Lambda_{6}m^{2}}-\mu_{2}a, \end{array}$$

where *g* is the signaling pathways from glucose to miR-451, *s* is the signaling pathways to AMPK complex, Λ_1_, Λ_3_ are the autocatalytic enhancement parameters for miR-451, AMPK complex, respectively, Λ_2_, Λ_4_ are the Hill-type inhibition saturation parameters from the counter part of miR-451 and AMPK complex, respectively, Λ_5_ is the inhibition strength of miR-451 by the AMPK complex, Λ_6_ is the inhibition strength of the AMPK complex by miR-451, *μ*_1_, *μ*_2_ are microRNA/protein degradation rates of miR-451 and AMPK complex, respectively. Table A1 in Appendix summarizes the dimensionless parameters. By taking the thresholds *th_M_* (=2.0) of miR-451 levels and *th_A_* (=2.0) of AMPK complex, we shall define the migratory region *M_m_* by *M_m_* = {(*M*, *A*) ∈ ℝ^2^: *M* < *th_M_*, *A* > *th_A_*} and the proliferative region *M_p_* by *M_p_* = {(*M, A*) ∈ ℝ^2^: *M* > *th_M_*, *A* < *th_A_*}. See Figure 2C for a diagram for proliferative and migratory regions.

## 3 Results

In this Section, we present analysis of the hybrid model and predictions for therapeutic strategies for eliminating invasive glioma cells.

### 3.1 Dynamics of the model

**Figure 3:**
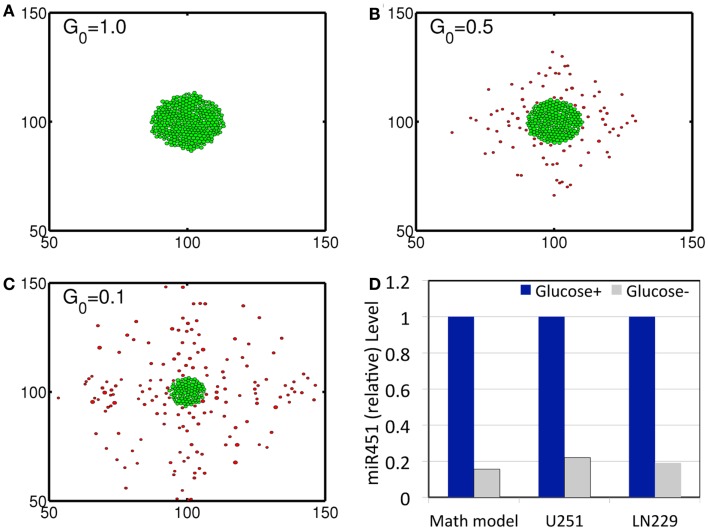
**(A–C)** Profiles of tumor spheroid in response to high (*G* = 1.0), intermediate (*G* = 0.5), and low (*G* = 0.1) glucose levels at *t* = 30 h. Domain size = [50 μm, 150 μm] × [50 μm, 150 μm] = [0.25, 0.75]^2^ in the dimensionless domain [0, 1]^2^). **(D)** Comparison between simulation results and experimental data. In response to high (*Glucose*+, blue) and low (*Glucose*−, gray) glucose levels, miR-451 expression levels are quantified. Simulation results are in good agreement with experimental results on U251 and LN229 cell line in Godlewski et al. (2010a). *λ_b_ = 0, $\lambda_{in}^{G}=0$. It was assumed that tumor cells respond to the glucose gradient for migration *in* *vitro* as in Godlewski et al. (2010a).

In order to validate the mathematical model, we first investigated invasion patterns of glioma cells embedded in high and low glucose levels in the absence of blood supply of glucose (*λ_b_* = 0), glucose injection ($\lambda_{in}^{G}=0$) and chemoattractants, and by assuming cells on the surface of the spheroid are migrating toward the glucose gradient (∆*G*) [i.e., by replacing $\frac{\nabla C}{\sqrt{K_{C}+|\nabla C|}}$ with $\frac{\nabla G}{\sqrt{K_{G}+|\nabla G|}}$ in the active force form in the equation (3)]. Figures 3A–C show spatial profiles of tumor spheroids in response to high (*G*_0_ = 1.0), intermediate (*G*_0_ = 0.5), and low (*G*_0_ = 0.1) glucose levels. Figure 3D shows relative miR-451 levels in response to high (*Glucose*+) and low (*Glucose*−) levels in simulations and experiments (U251 and LN229 cell lines) (Godlewski et al., 2010a). General patterns of tumor spheroids in response to high (Figure 3A) and low (Figure 3C) glucose levels in simulations are in good agreement with experimental observations in Godlewski et al. (2010a) where high (4.5 g/l) and low (0.3 g/l) levels of glucose induced over- and under-expression of miR-451 (see Figure 3D), leading to proliferating (as in Figure 3A) and dispersed invasive (as in Figure 3C) patterns of tumor cells, respectively.

**Figure 4:**
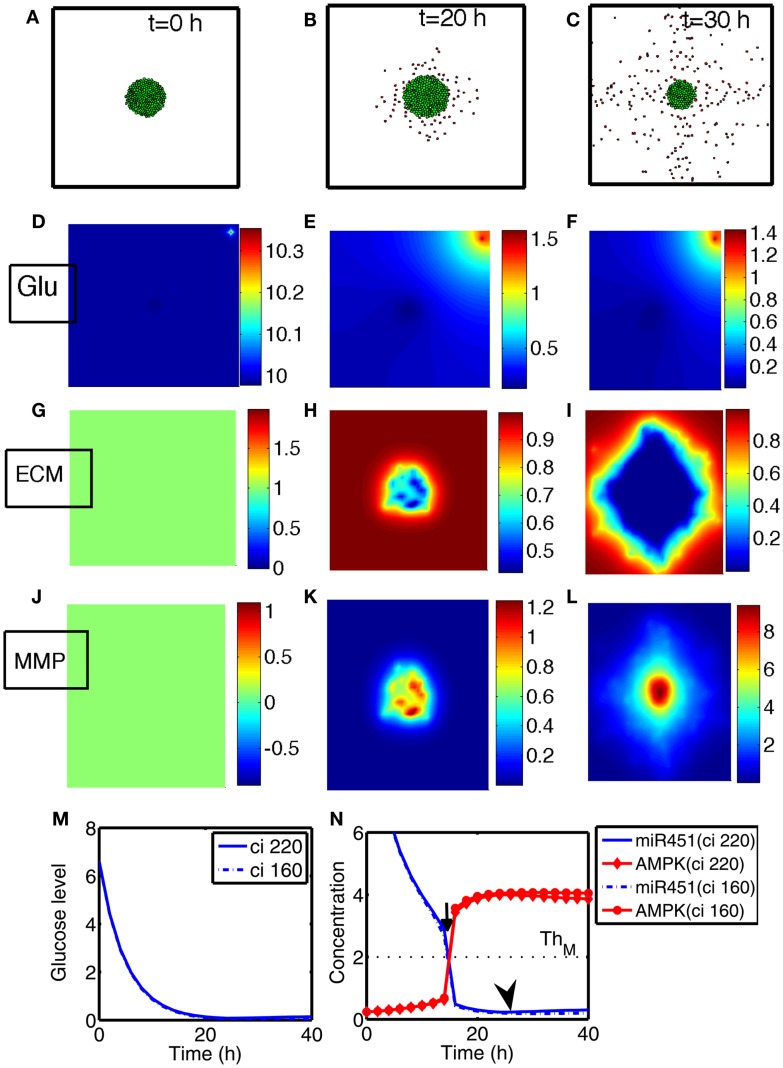
**(A–C)** Tumor invasion pattern at time *t* = 0, 20, 30 h in response to glucose levels (*G*_0_ = 10). Domain size = [0.25, 0.75] ⊂ [0, 1]^2^. **(D–F)** Profiles of glucose concentration on the domain [0, 1] × [0, 1]. Glucose flux from a blood vessel induces a peak value at the upper right corner. **(G–I)** Profiles of ECM on the subdomain [0.25, 0.75] × [0.25, 0.75] ⊂ [0, 1]^2^. ECM were degraded in the invasive region. **(J–L)** Profiles of MMPs on the subdomain [0.25, 0.75] × [0.25, 0.75] ⊂ [0, 1]^2^. MMPs are localized in tumor region. **(M)** Glucose levels at two cell sites (cell id = 220, 160). **(N)** Concentrations of intracellular variables, miR-451 and AMPK complex, at the cell sites in **(M)**.

We investigate invasion dynamics of a growing tumor in response to glucose levels in the presence of blood supply. Figures 4A–C show spatial patterns of a growing/invading tumor in response to glucose supply (*G*_0_ = 10) at time *t* = 0, 20, 30 h. Initial high glucose level is decreased due to glucose consumption by tumor cells at the center of the domain and nearby tissue (Figures 4D–F). The miR-451 activity at cell sites is decreased and AMPK levels creep up due to decreased glucose levels (Figures 4M,N). Cells on the surface of the tumor mass immediately respond to intracellular biochemical signals (miR-451 and AMPK levels) and begin to migrate when miR-451 level drops below the threshold (*M* < *th_M_* = 2.0) and AMPK level is up-regulated (*A* > *th_A_* = 2.0) due to decreased glucose level at the cell site (cf. Figure 2C). However, cells at the center of the tumor mass are surrounded by neighboring cells and stay inside the tumor due to physical constraints despite biochemical migration signals (*M* < *th_M_*, *A* > *th_A_*). Figure 4N shows time courses of miR-451 activities and AMPK levels in response to glucose levels in Figure 4M at two cell sites [cell id = 220 with initial location (108.2, 99.5); cell id = 160 with initial location (100.5, 99.8)]. The miR-451 levels for both cells drop below the threshold (*th_M_*) and AMPK levels are above the threshold (*th_A_*) around *t* = 16 h already, generating “migratory” signal. However, the cell (marked in “gray”; cell id = 220) in Figure 4A still stays in the tumor mass at *t* = 20 h (Figure 4B) and begins to shed off at later time (*t* = 26 h; arrow in Figure 4N) when free space is available. Another cell (cell id = 160) at the center of domain remains at the center of the tumor due to physical constraints until final time [*t* = 40 h; final location (100.3, 99.7)]. When glioma cells on the surface of the tumor mass migrate into surrounding brain tissue, proteinases (MMPs) are secreted in the invading front and ECM is degraded due to high levels of MMPs (proteolysis). See localized MMPs at cell sites and degraded ECM profiles in Figures 4G–L, respectively.

**Figure 5:**
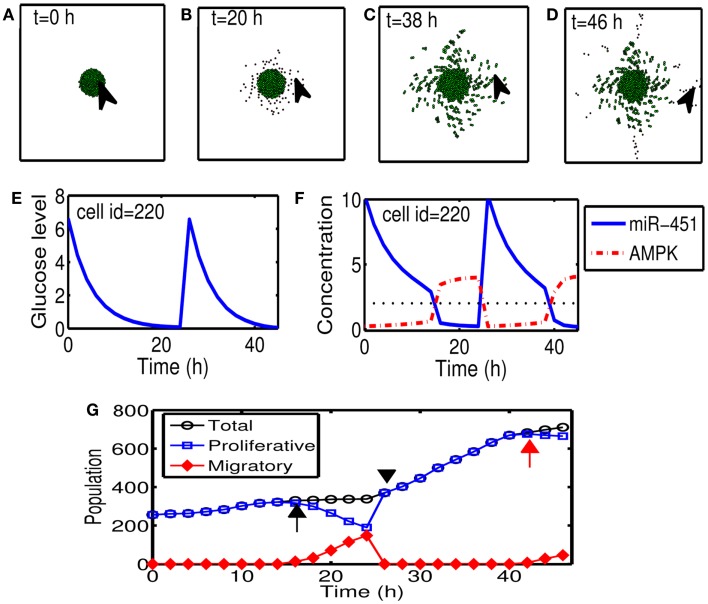
**(A–D)** Tumor growth patterns at *t* = 0, 20, 38, 46 h in response to glucose injection. Two cycles of proliferation-migration pattern are observed after initial (*t* = 0 h) and second (*t* = 26 h) injections of glucose (*G* = 10). Domain size = [0.2, 0.8] × [0.2, 0.8] ⊂ [0, 1]^2^. **(E)** Glucose level at a cell site [cell id = 220; arrowhead in **(A–D)**]. **(F)** Time course of miR-451 activity and AMPK concentration at the cell site in **(E)**. Dotted black line in the middle = threshold value of miR-451 (*th_M_*). **(G)** Time course of cell populations: proliferative (blue square), migratory (red diamond), and total (black circle) cells. Some of proliferative cells become migratory ones around *t* = 16 h (black arrow) and *t* = 42 h (red arrow) when miR-451 levels drop below threshold due to lowered glucose levels. All migratory cells enter the proliferative phase around *t* = 26 (black arrowhead) in response to glucose injection.

Figures 5A–D show cyclic tumor growth patterns at *t* = 0, 20, 38, 46 h in response to periodic glucose injection. When high doses (*G* = 10.0) of glucose are introduced into the system at *t* = 0, 26 h, fluctuating glucose values (Figure 5E) at a cell site (cell id = 220; arrowhead in Figures 5A–D) lead to a cycle of up-regulation and down-regulation of miR-451 (Figure 5F). Cells outside the tumor core respond to this stimulus by either proliferating or migrating. See Figures 5A–D. Cell phenotype changes between proliferative and migratory cells are more clear in Figure 5G. Initial proliferative cells due to high glucose levels change their phenotype to become migratory cells whenever glucose level lowered to induce migratory phase [*M* > *th_M_*, *A* < *th_A_*; ∼*t* = 16 h (black arrow) and *t* = 42 h (red arrow)]. These migratory cells change their phenotypes to proliferative cells (∼*t* = 26 h; black arrowhead) when the high glucose level from glucose injection induces proliferative phase (*M* > *th_M_*, *A* < *th_A_*).

### 3.2 Sensitivity of the model to inhibition parameters (*α*, *β*) in the core system

**Figure 6:**
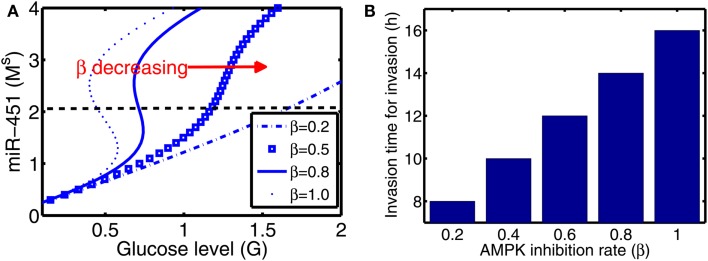
**(A)** Steady state values of miR-451 (*M^s^*) as a function of glucose level (*G*) for various inhibition strength of AMPK by miR-451 (*β*) in core control system. **(B)** Effect of the inhibition rate (*β*) on activation time for glioma cell invasion in the hybrid model. As *β* is decreased, activation time for invasion is decreased. See Appendix A.2 for definition and role of *β* in the core system.

From now on, the *activation* *time* *for invasion* is defined to be time when a cell in proliferative phase (*M* > *th_M_*, *A* < *th_A_*) changes its phenotype to a migratory cell (*M* < *th_M_*, *A* > *th_A_*) due to a microenvironmental change (glucose fluctuation) and begins to migrate away from the main tumor aggregate for the first time among all other cells. In Figure 6A we show steady state values of miR-451 (*M^s^*) in response to different glucose levels for various inhibition strength (*β*) of AMPK complex by miR-451. See Appendix A.2 for definition and role of *β* (and *α* below) in the core system. As *β* is decreased the bifurcation curve shifts to the right (higher glucose levels). In the control case (*β* = 1.0), a relatively low glucose level (*G* = 0.4) is required for activation of cell invasion. When this inhibition strength is weakened (*β* small), a cell may begin to migrate for larger glucose levels. Figure 6B shows activation time for invasion as a function of inhibition strength of AMPK complex production (*β*). As *β* is decreased from control base parameter (*β* = 1.0), the rate of AMPK complex formation is increased and miR-451 level is decreased at earlier time, leading to early activation time for invasion. This might have biological implications. For example, one could totally use or partially block inhibition pathways from miR-451 to AMPK (decrease in *β*) in order to boost invasion activation under certain circumstances.

**Figure 7:**
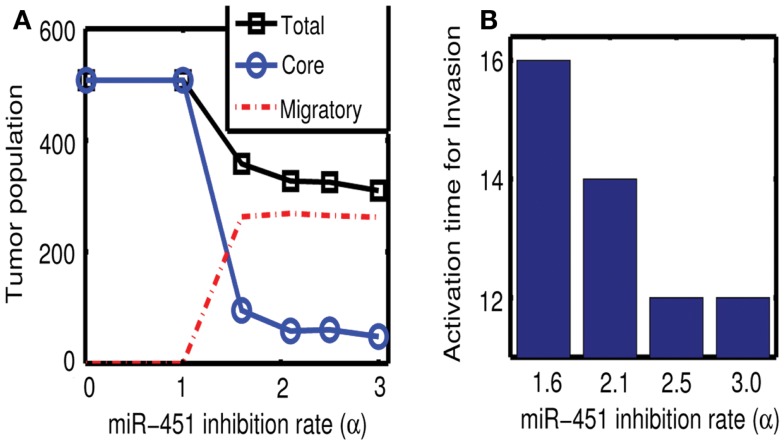
**(A)** Effect of miR-451 inhibition strength (*α*) on tumor population. As *α* is increased, miR-451 production rate is decreased leading to low miR-451 activity and activation of glioma cell migration. **(B)** Effect of *α* on activation time for cell invasion. As *α* is increased, lowered miR-451 activity leads to early activation of cell invasion. See Appendix A.2 for definition and role of *α* in the core system.

Figures 7A,B show effect of inhibition strength (*α*) of miR-451 production by AMPK complex on tumor population and activation time for cell invasion. As the inhibition strength *α* is increased, miR-451 levels are decreased at earlier time (Figure 7B) and more cells on the surface of the tumor mass migrate from the biochemical signal. See Figure 7A. This would have several implications. For example, cell migration or dispersion might be prevented by any drug that blocks inhibitory activity of AMPK complex to miR-451. This may generate faster growth of tumor mass since most of cells would be in proliferative phase. Therefore, this could be used as a temporary way of holding cell migration in order to not miss out single migratory cells for surgery.

### 3.3 Predictions of the model for a possible therapeutic approach

**Figure 8:**
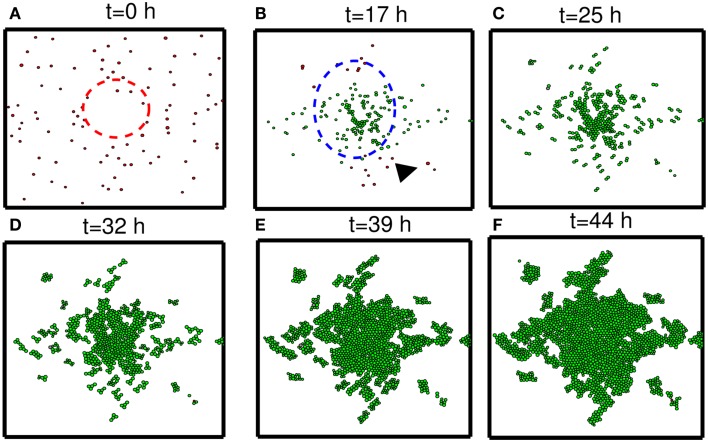
**Strategy of eradicating migratory cells after first surgery**. **(A–F)** Spatial profile of a surgically removed tumor at *t* = 0, 17, 25, 32, 39, 44 h. Detectable tumor core was surgically removed at *t* = 0 h (red dotted circle) and a chemoattractant was injected at the center of the removed area immediately after surgery (*t* = 0 h). After waiting 17 h, glucose was injected at the center of the removed area again in order to turn the migratory switch (*M* < *th_M_*, *A* > *th_A_*) off and make these cells grow (*M* > *th_M_*, *A* < *th_A_*). This growing mass of tumor may be *visible* for secondary surgery, leading to eradication of *invisible* migratory cells. Domain size = [0.25, 0.75] × [0.25, 0.75] ⊂ [0, 1]^2^.

**Figure 9:**
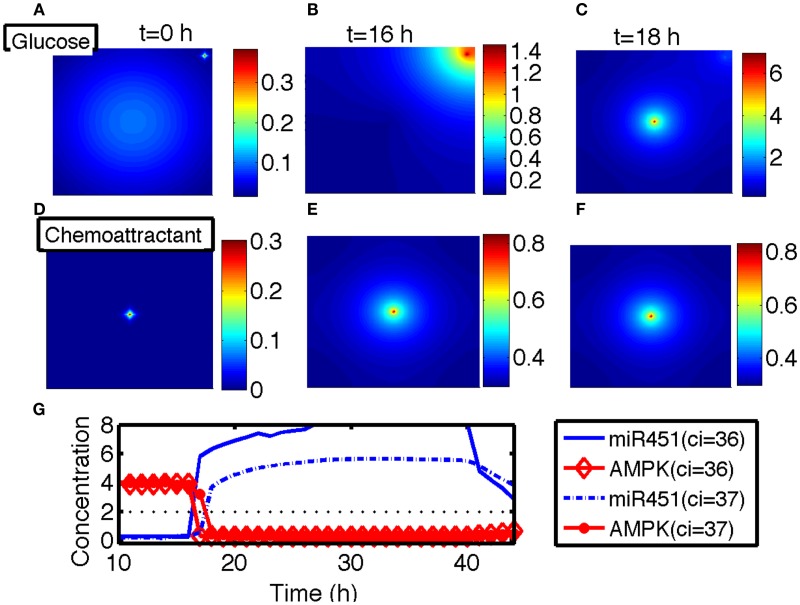
**(A–C)** Spatial profiles of glucose at *t* = 0, 16, 18 h. Glucose was injected at the center of the domain around *t* = 17 h. Glucose flux from a blood vessel induces a peak value at the upper right corner. **(D–F)** Spatial profiles of chemoattractant at *t* = 0, 16, 18 h. Chemotactic source was located at the center of the domain. **(G)** Time course of the miR-451 activity and AMPK level at two cell sites (cell id = 36, 37). *Domain size in **(A–F)** = [0, 1]^2^.

In this section, we developed therapeutic strategies for eradicating *invisible* migratory glioma cells in the brain after conventional surgery where only *visible* parts of tumor mass are removed. Here we assume that invasive cells in surrounding tissue can sense and respond to the chemoattractant gradient (∆*C*). Figures 8A–F show spatial profiles of the tumor cells at *t* = 0, 17, 25, 32, 39, 44 h, respectively. After first surgery (region inside red doted circle in Figure 8A) at *t* = 0, a chemoattractant was injected at the center of the resected area. See Figures 9D–F for spatial profiles of chemoattractant at time *t* = 0, 16, 18 h. Invasive cells begin to migrate back to the surgical site. After waiting 17 h ($t_{1}^{G}=17$), a high dose of glucose was introduced into the system at the center of surgical site and glucose molecule diffuses through the domain (Figures 9A–C). High glucose levels trigger the intracellular switch from the migratory phase to the proliferative mode for invasive cells near the injection site (Figure 9G). Figure 9G shows time courses of miR-451 activity and AMPK level at two cell sites (cell id = 36, 37). A cell (cell id = 36) close to the center of the resection site is activated for proliferation at the earlier time (∼*t* = 17). Green cells inside the blue dotted circle in Figure 8B represent proliferative cells due to high glucose levels while red cells outside the blue circle are still migratory cells in migratory phase (*M* < *th_M_*, *A* > *th_A_*) from low glucose levels. While infiltrative tumor cells after surgery may not be detected, regrown tumor mass may be detected by conventional screening tools such as MRI when tumor density is high enough. This may increase the probability of eliminating invasive cells.

**Figure 10:**
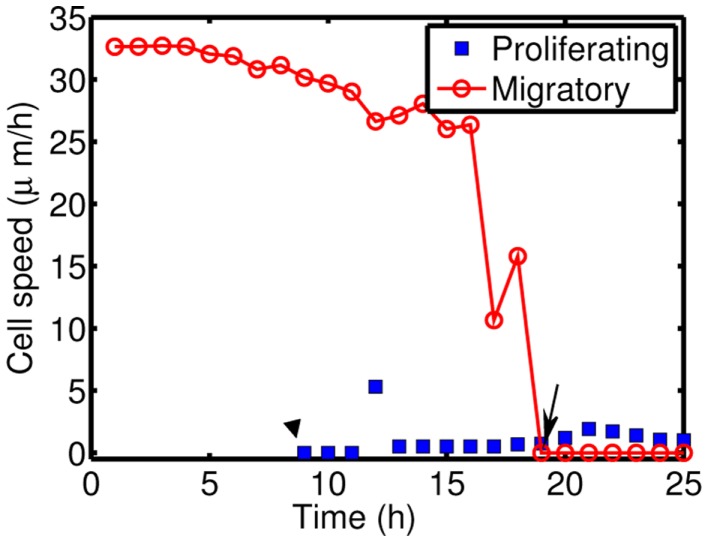
**Cell speeds of proliferative (blue square) and migratory (solid circle, red) cells in Figure 8**.

Figure 10 shows time courses of average cell speeds of migratory (solid circle, red) and proliferative (blue square) cells in Figure 8. From the beginning of the simulation, the average cell speed of cells in migratory phase maintained the values in the rage of 27–33 μm/h until cell speeds begin to drop down around ∼17 h due to cell aggregation at the center of domain. Eventually all migratory cells become proliferative cells around *t* = 19 h. Proliferative cells (blue square, transformed from migratory cells around 9 h) show very low cell speeds due to absence of active force since their movement is due to growth not cell movement. Cell speeds have been reported to be in the range of 39–45 μm/h in 2D barrier-free culture condition and 15–20 μm/h in 3D glioblastoma cell culture in the absence/presence of EGF-stimulation (Kim et al., 2008), 15–25 μm/h in glioblastoma cells with/without *α*-actinin isoforms (Sen et al., 2009), 15–48 μm/h for cells embedded in collagen I matrix (Kaufman et al., 2005). So, the cell speed in our model is in good agreement with experimental data.

**Figure 11:**
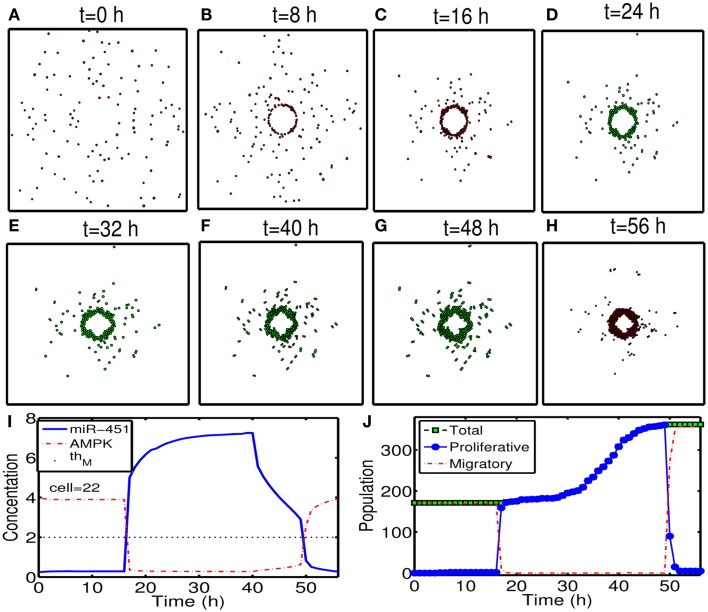
**(A–H)** Tumor migration-proliferation patterns at *t* = 0, 8, 16, 24, 32, 40, 48, 56 h in response to injection of a chemoattractant at *t* = 0 h and glucose at *t* = 17 h after initial surgery at *t* = 0 h. Migratory cells switch to proliferative phenotype, forming a *visible* larger tumor mass, in response to glucose for second follow-up surgery. Migratory cells stop on the periphery of the resected area from the first surgery. It was assumed that a cell can sense the environment of the resection bed and the active force of a migratory cell is set to be zero when the cell reaches the periphery of the resection bed. Domain size = [0.2, 0.8] × [0.2, 0.8] ⊂ [0, 1]^2^. **(I)** Time course of miR-451 activity and AMPK level at a cell site (cell id = 22). Dotted black line in the middle = threshold value of miR-451 (*th_M_* = 2.0). **(J)** Time course of cell populations: proliferative (blue circle), migratory (red dotted), and total (green square) cells. All migratory cells switch to proliferative ones around *t* = 17 h when low miR-451 levels jump to higher value [*M* > 5 in **(I)**] and the level stays above threshold (*thM* = 2.0) due to glucose injection. Most of these proliferative cells enter the migratory phase (*M* < *th_M_*, *A* > *th_A_*) again around *t* = 49 h due to lowered glucose levels. Parameters used: $t_{1}^{G}=17\;\text{h, }\tau_{d}^{G}=24\;\text{h}\text{.}$

To test our hypothesis in a more realistic situation, we tested our hypothesis of attracting cancer cells back to the resection bed in a more realistic setting in Figure 11. Here we assume that a cell can sense the environment of the resection bed and stop moving on the edge of the resection bed. In our simulation, generation of active force of a migratory cell is turned off, i.e., active force **T**_i_ in the equation (3) is set to be zero when the cell reaches periphery of the resection bed. The simulation begins immediately after initial surgery of the large tumor mass at the center of the domain again. Figures 11A–H show proliferation and migration patterns of tumor cells at *t* = 0, 8, 16, 24, 32, 40, 48, 56 h in response to initial injection of a chemoattractant (*t* = 0 h) followed by glucose injection at *t* = 17 h. Figures 11I,J show time courses of miR-451 activity and AMPK level at a cell site (cell id = 22) and cell populations [proliferative (blue circle), migratory (red dotted), and total (green square) cells], respectively. Initially, there exist only a phenotype of migratory cells but these cells begin to aggregate on the periphery of the resection bed. However, these cells switch their phenotype to proliferative ones via core control system (Figure 11I) in response to glucose injection at *t* = 17 h. These proliferative cells form a *visible* larger tumor mass which can be ready for the second follow-up surgery. Most of these proliferative cells enter the migratory phase (*M* < *th_M_*, *A* > *th_A_*) again around *t* = 49 h due to lowered glucose levels.

### 3.4 Therapeutic optimization

**Figure 12:**
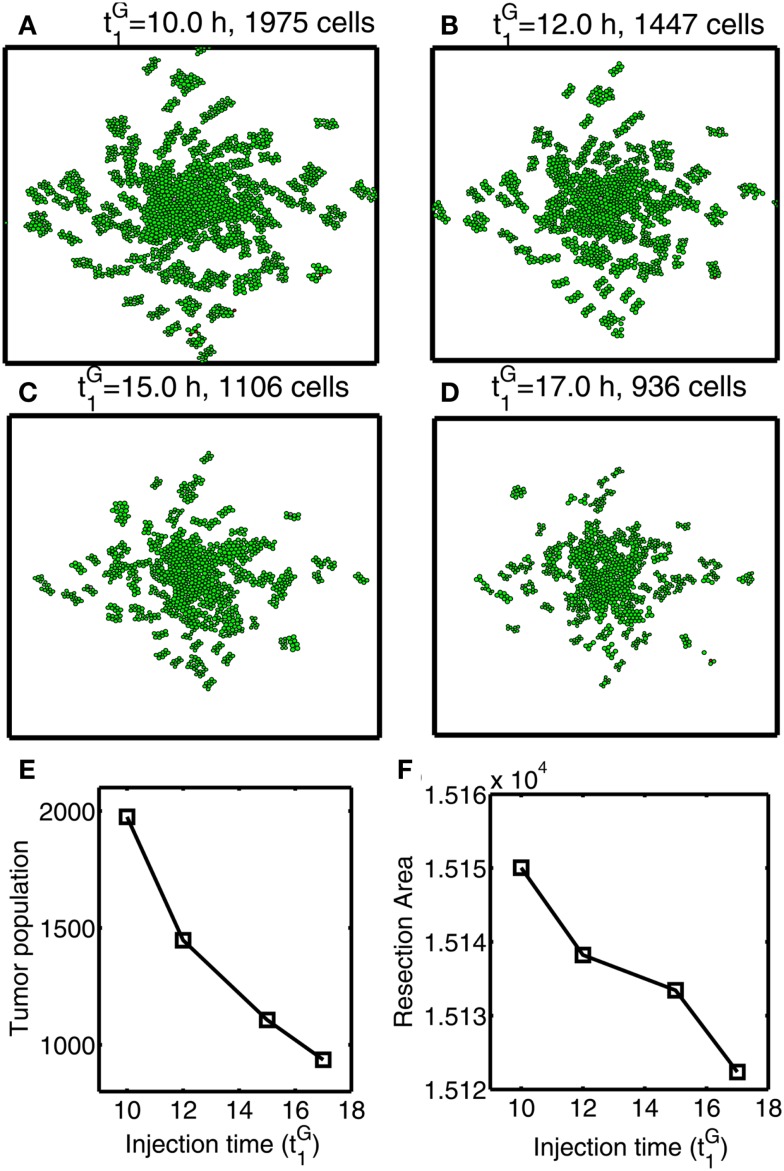
**Optimal strategy for glucose injection for second surgery after attracting invasive cells via chemotaxis**. **(A–D)** Growth patterns at final time (*t* = 33 h) for various glucose injection time $\left(t_{1}^{G}=10\;\left(A\right),12\left(B\right),15\;\left(C\right),17\;h\left(D\right)\right).$ Domain size = [0.2, 0.8] × [0.2, 0.8] ⊂ [0, 1]^2^. **(E)** Tumor population for different $t_{1}^{G}.$ Tumor population is decreased as $t_{1}^{G}$ is increased due to delay of growth signal. **(F)** Resection area for second surgery for various $t_{1}^{G}.$ Resection area is decreased as injection time is increased.

In Figures 12A–F, we investigate the effect of glucose injection time on tumor growth patterns for chemoattractant-induced second surgery under same conditions as in Figure 8. Figures 12A–D illustrate spatial growth patterns of tumor cells at final time (*t* = 44 h) when a high dose of glucose was injected at the center of the surgical site at different initial injection time ($t_{1}^{G}=10,\;12,\;15,\;17\;h$). When glucose was injected at the earlier time ($t_{1}^{G}=10\;h$), more invasive cells enter the cell cycle (*M* > *th_M_*, *A* < *th_A_*) for higher flux of glucose before they reach the surgical site. For the case of glucose injection at later time ($t_{1}^{G}=17\;h$), more cells are localized at the center of the surgical site. Figure 12E shows tumor population at final time. Cells inside the bigger size of tumor mass are subject to slower growth (see mechanical aspects of the hybrid model). Initial glucose injection time decreases tumor population since more tumor cells are activated for proliferation and tumor cells grow faster without physical constraints. This injection time also decreases resection area for second surgery due to localized tumor cells at the original surgical site (Figure 12F). Therefore, choosing appropriate injection time is important for efficacy of tumor treatment. However, any further delay of second surgery would lead to larger size of tumor mass and one may face another possibility of tumor invasion.

**Figure 13:**
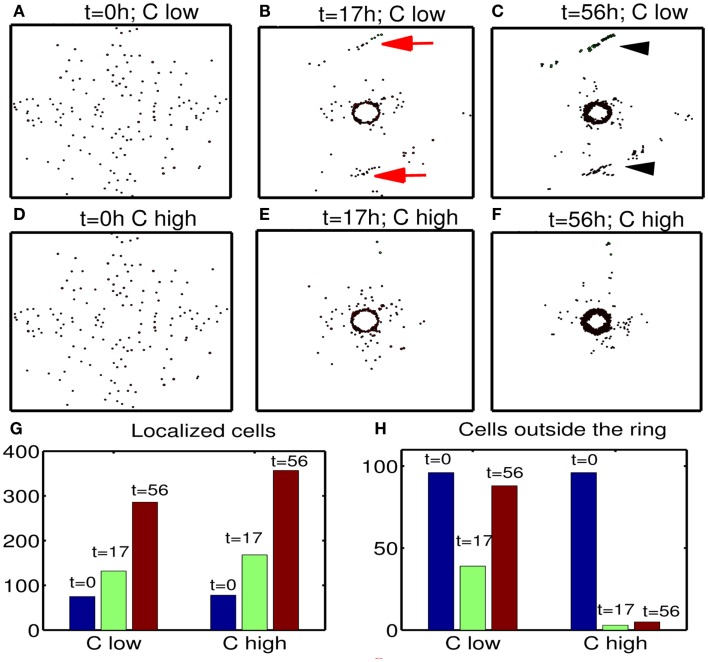
**Effect of chemoattractant strength ($\lambda_{in}^{C}$) on efficacy of the therapy**. **(A–F)** Tumor migration-proliferation patterns for low (${\bar{\lambda }}_{in}^{C}=\lambda_{in}^{C∕10}$) and high (control) levels of a chemoattractant at *t* = 0, 17, 56 h. A high dose of glucose was injected at *t* = 17 h after initial surgery at *t* = 0 h. When the chemoattractant level is low, cells in the far away field do not effectively respond to the chemoattractant (red arrows in **(B)**), which induces further growth later at the undesirable location (black arrowheads in **(C)**). Domain size = [0.1, 0.9] × [0.1, 0.9] ⊂ [0, 1]^2^. (G) Populations of localized cells (cells with *d* < 0.25) for low (10-fold smaller) and high levels of chemoattractant. Here, $d=\sqrt{{\left(x_{i}-0.5\right)}^{2}+{\left(y_{i}-0.5\right)}^{2}}$ is the distance from cell location (*x_i_*, *y_i_*) to the center (0.5, 0.5) of the domain. **(H)** Same as in **(G)** but for cells outside the localized domain (cells with *d* > 0.25). Parameters used: $t_{1}^{G}=17\;\text{h, }\tau_{d}^{G}=24\;\text{h}\text{.}$

Next, we investigate the effect of chemoattractant strength on efficacy of therapeutic strategies suggested in this paper. Figures 13A–F show migration-proliferation patterns of tumor cells at *t* = 0, 17, 56 h in the presence of low (${\bar{\lambda }}_{in}^{C}=\lambda_{in}^{C∕10}$) and high (control) levels of a chemoattractant and glucose injection at *t* = 17 h after initial surgery at *t* = 0 h. When the chemoattractant level is low, cells in the far away field do not effectively respond to the chemotactic signals (red arrows in Figure 13B) and do not move toward the resection bed easily, which induces further tumor growth later at the undesirable location (black arrowheads in Figure 13C). Figures 13G,H show populations of localized cells (cells with *d* < 0.25) and cells outside the localized domain (cells with *d* > 0.25), respectively. Here, $d=\sqrt{{\left(x_{i}-0.5\right)}^{2}+{\left(y_{i}-0.5\right)}^{2}}$ is the distance from cell location (*x_i_*, *y_i_*) to the center (0.5, 0.5) of the domain. One can see that the low chemoattractant level leads to a moderate decrease in the population of localized cells (Figure 13G) but induces a significant increase in the population of cells in the far away field (Figure 13H) at *t* = 17, 56 h. These missed cells reduce efficacy of second surgery. These results effectively show that enough levels of chemoattractant need to be provided for the more efficient therapy.

## 4 Discussion

One major challenge for treating glioblastoma is that by the time the disease is diagnosed cancer cells have already invaded other parts of the brain, inhibiting complete elimination of cancer cells. Blocking this critical invasion process or finding a way of eradicating invasive tumor cells would lead to better clinical outcomes. Godlewski et al. (2010a) recently identified a key miRNA, miR-451, and its target, AMPK complex, that regulates a critical switch between cell migration and proliferation. Reduced miR-451 activity has been associated with cancers (Bandres et al., 2009) including glioma (Gal et al., 2008; Godlewski et al., 2010a). In the harsh microenvironment, glioblastoma cells shift their metabolic machinery toward a high level of glucose uptake, *Warburg* *effect* (Warburg, 1956; Kim and Dang, 2006; Heiden et al., 2009), and lowered glucose levels trigger active cell migration toward the better microenvironment. Some up-regulated miRNAs in brain tumors are believed to play a pro-oncogenic role via supporting growth, proliferation, migration, and survival of cancer cells while expression of other miRNA having anti-tumor effects is suppressed in gliomas. These miRNAs harbor a therapeutic significance as therapeutic agents in anti-cancer therapy (Lawler and Chiocca, 2009; Godlewski et al., 2010b; Chistiakov and Chekhonin, 2012). Godlewski et al. (2010a) illustrated glucose regulation of proliferation and migration of glioma cells: (i) low glucose ⇒ down-regulation of miR-451 and up-regulation of the AMPK complex ⇒ cell migration (ii) normal (high) glucose ⇒ up-regulation of miR-451 and down-regulation of AMPK complex ⇒ proliferation.

Conventional treatment options such as radiotherapy and chemo therapy after surgical resection of tumor mass lead to poor clinical outcome in many cases of glioblastoma due to invisible migratory cancer cells in the brain tissue. In the present paper we aimed at understanding a basic mechanism of proliferation and migration of glioma cells in response to fluctuating glucose levels and developing therapeutic strategies for eradicating *invasive* glioma cells after initial conventional surgery. The present paper develops a hybrid mathematical model of glioma cell migration and proliferation. The hybrid model considers proliferation and migration of individual tumor cells based on cell-mechanics, concentrations of glucose, chemoattractant, ECM, and MMPs in a spatio-temporal domain, and regulation of key intracellular molecules, miR-451 and AMPK complex, at each cell site. Mechanical stress and active forces acting on tumor cells were taken into account in the model. The spatial distribution of both proliferative and migratory cells in response to high and low glucose levels is in good agreement with experiments (Godlewski et al., 2010a).

We first considered the important role that the core control system (miR-451, AMPK) plays in regulation of the migratory phase and proliferative phase when cells on the surface of tumor mass begin to migrate away from the main core in the harsh microenvironment where glucose levels fluctuate. We analyzed the migratory behavior of cells in response to variations in two key parameters, inhibition strength of miR-451 (*α*) and inhibition strength of AMPK complex (*β*), that play a critical role in characterizing the invasion of glioma cells. Active migration of a cell depends on chemical signals from the core miR-451-AMPK system and their physical microenvironment in response to glucose injection. Growing tumor cells in the presence of abundant glucose switch their mechanism for cell migration when glucose is not available and cells are not subject to physical constraints. When glucose was introduced into the system in a periodic fashion, tumor repeat migration-proliferation cycle, which may lead to faster growth (Kim et al., 2011a).

For therapeutic strategies, the model suggested that (i) Introduction of chemoattractant at the surgical site may bring these invasive tumor cells back to the tumor site. (ii) Glucose injection at the center of the surgical site would lead to up-regulation of miR-451 and down-regulation of AMPK complex, which induces cell proliferation. (iii) Follow-up surgery may eradicate the tumor cells that managed to survive from the first surgery. Multiple microsurgical resections for glioblastoma have been proven to be effective and useful (Hong et al., 2012). However, we also found that glucose injection at the wrong time may grow the tumor even before tumor cells gather together and this may lead to undesirable results, faster growth of dispersed tumor mass. Detecting appropriate time glucose injection and second surgery might also be a challenge. Tumor cells can be cultured from biopsies up to 4 cm away from the bulk tumor (Silbergeld and Chicoine, 1997). When cancer cells migrate too far from the original site, it may be difficult to attract these cells. For example, we found that too low chemoattractant may not be able to attract all migratory cells for second surgery (Figure 12) and these *missed* cells would decrease efficacy of the therapy. Therefore, in order to attract those invasive cells in the far away field (>4 cm away) one might need strong chemoattractants at the resection bed. However, this new strategy in this paper may be a novel way of eliminating all cancer cells when an appropriate combination of chemoattractants and glucose is used.

The analysis and predictions of the hybrid model in this paper may serve as a starting point for experimentation and more detailed modeling. We indicate several aspects and directions for further development of *in* *vivo* and/or *in* *vitro* multi-scale models: (i) One might use a multi-phase approach to describe the inhomogeneity of the microenvironment (Byrne and Preziosi, 2004; Preziosi and Tosin, 2009; Preziosi and Vitale, 2011). See a review (Lowengrub et al., 2010) for further discussion. (ii) It has been suggested that some glioma cells may migrate while they grow. We could incorporate this aspect easily in this hybrid framework. Collective cell migration is also considered as a key aspect of tumor invasion (Friedl and Alexander, 2011). Creating a microtrack of locally digested ECM followed by generating a larger excavated macrotrack by proteolysis was suggested as a way of collective cell migration (Wolf et al., 2007; Friedl and Alexander, 2011; Ilina et al., 2011). In recent study, Sampetrean et al. (2011) also illustrated the importance of collective migration along fiber tracts in glioma cell invasion, suggesting the need for anti-invasion approach. (iii) The simplified network of miR-451 and AMPK complex can be extended to the more detailed network in order to take into account cell cycle and other anti-cancer molecules. (iv) Some important players such as immune cells and cytokines in the microenvironment should be included in the model (Cheng and Weiner, 2003; Rejniak and McCawley, 2010; Wiranowska and Rojiani, 2011). (v) It was observed that isoforms of myosin II are specifically required for an adaptation needed to squeeze through the dense network of other cells (Beadle et al., 2008). Detailed modeling work is necessary to take into account deformation of cell body for cell motility. We hope to address these issues in future work.

## Conflict of Interest Statement

The authors declare that the research was conducted in the absence of any commercial or financial relationships that could be construed as a potential conflict of interest.

## A Appendix

### A.1 Parameter estimation and non-dimensionalization

#### A.1.1 Tumor module

The characteristic distance is the maximum domain size (*L* = 2 mm) of growing glioblastoma within 48 h similar to the size of the migratory region in the experiments by Godlewski et al. (2010a) and we use *T* = 1 h to get dimensionless variables and parameters:

(A1) $$\begin{array}{c} \bar{t}=\frac{t}{T},\bar{x}=\frac{x}{L},Ḡ=\frac{G}{G^{*}},\bar{C}=\frac{C}{C^{*}},\bar{\rho }=\frac{\rho }{\rho^{*}},\bar{P}=\frac{P}{P^{*}},\;\\ {\bar{D}}_{G}=\frac{T}{L^{2}}D_{G},{\bar{D}}_{C}=\frac{T}{L^{2}}D_{C},\\ {\bar{D}}_{P}=\frac{T}{L^{2}}D_{P},{\bar{\lambda }}_{in}^{G}=\frac{T\lambda_{in}^{G}}{G^{*}},{\bar{\lambda }}_{b}=\frac{T\lambda_{b}}{G^{*}},{\bar{\lambda }}_{c}=\frac{T\lambda_{c}}{G^{*}},\;\\ {\bar{\lambda }}_{in}^{C}=\frac{T\lambda_{in}^{C}}{G^{*}},{\bar{\lambda }}_{1}=T\lambda_{1}P^{*},\\ {\bar{\lambda }}_{2}=T\lambda_{2},{\bar{\rho }}_{*}=\frac{\rho_{*}}{\rho^{*}},{\bar{\lambda }}_{3}=T\lambda_{3},{\bar{\mu }}_{G}=T\mu_{G},\;\\ {\bar{\mu }}_{C}=T\mu_{C},{\bar{\mu }}_{P}=T\mu_{P}, \end{array}$$

We estimate some of parameters and reference values in the following. (i) *D_G_*: diffusion coefficients of glucose (*G*) were measured to be 6.7 × 10^−7^ cm^2^/s in the brain (Jain, 1987) and 1.3 × 10^−6^ cm^2^/s in collagen gel (Rong et al., 2006). We take *D_G_* = 6.7 × 10^−7^ cm^2^/s as in Jain (1987). (ii) *D_P:_* in experiments of the movement of MMP-1in the collagen fibril, Saffarian et al. (2004) estimated the diffusion coefficient of MMPs as (8.0 ± 1.5) × 10^−9^ cm^2^/s for inactive mutant and (8.0 ± 1.5) × 10^−9^ cm^2^/s for wild-type activated MMP-1, respectively. For our simulation, we take *D_P_* = 8.0 × 10^−9^ cm^2^/s. (iii) *D_C_*: the diffusion coefficient of chemoattractant was taken from one of EGF, *D_C_* = 1.66 × 10^−6^ cm^2^/s from Thorne et al. (2004). (iv) *λ_c_* (glucose consumption rate): the measured level *α* = 1.6 pg/cell/min for piecewise increasing linear consumption term *α*(*G*)*n* was used in a glioma invasion study (Sander and Deisboeck, 2002) along with a threshold value $G_{1}^{th}=2.0\times 10^{-4}\;\text{g/c}{\text{m}}^{\text{3}},$ taken from the work of Li (1982). We take *λ_c_* = 0.8 pg/cell/min and use a sphere with a radius *r* = 8–20 μ*m* for estimation of cell volume. (v) *G**: Sander and Deisboeck (2002) used the characteristic concentration of glucose 2 × 10^−4^ g/cm^3^, and took the value 6 × 10^−4^ g/cm^3^ for glucose level far from the tumor (see also Deisboeck et al., 2001). Kim et al. (2009) took 6 × 10^−4^ g/cm^3^ as a reference value based on experimental observation. In *in vitro* experimental study (Godlewski et al., 2010a), high (4.5 g/l = 4.5 × 10^−3^ g/cm^3^) and low (0.3 g/l = 3.0 × 10^−4^ g/cm^3^) levels of glucose induced the up- and down-regulated miR-451 expression. We take the high glucose level, *G** = 4.5 × 10^−3^ g/cm^3^, as a reference value. (vi) ρ*: collagen is one of main ECM components. ECM concentration was estimated to be 0.5–2.0 mg*/*ml in an experimental study of growth patterns of glioma spheroids. Stein et al. (2007) investigated growth patterns of glioma cell lines in experiments where U87 wild-type and its mutant, U87DEGFR, were implanted into collagen I of concentration of 2.6 mg*/*ml. We take ρ* = 1.0 mg/cm^3^ as our reference value of ECM. (vii) *P**: in a breast cancer cell invasion study, MMP concentrations were measured to be 1.6 × 10^−9^ g/cm^3^ (Jia et al., 2004). On the other hand, it was shown that PCK3145 can down-regulate MMP-9 level in prostate cancer patients with up-regulated MMP-9 level of >100 μg/l (Annabi et al., 2005). We take the high levels of MMPs (*P** = 100 μg/l) as our reference value of MMPs.

Table 1 lists reference values and all parameter values above.

**Table A1:** **Parameters that are used in the intracellular miR-451-AMPK system**.

	Description	Value	Refs.
*l_g_*	Glucose signaling rate	1.0	Kim et al. (2011a)
*l*_1_	Autocatalytic production rate of miR-451	4.0	Kim et al. (2011a)
*l*_2_	Hill-type coefficient	1.0	Kim et al. (2011a)
*α*	Inhibition strength of miR-451 by AMPK complex	1.6	Kim et al. (2011a)
*th_M_*	Threshold of AMPK for proliferation/migration switch	2.0	Estimated
*l*_3_	Autocatalytic production rate of AMPK	4.0	Kim et al. (2011a)
*l*_4_	Hill-type coefficient	1.0	Kim et al. (2011a)
*β*	Inhibition strength of AMPK complex by miR-451	1.0	Kim et al. (2011a)
*S*	Signaling source of AMPK	0.2	Kim et al. (2011a)
*∈*	Scaling factor (slow dynamics) of AMPK complex	0.02	Crute et al. (1998); Aguda et al. (2008); Gantier et al. (2011); Kim et al. (2011a)
*th_A_*	Threshold of AMPK for proliferation/migration switch	2.0	Estimated

##### A.1.2 Core control system (miR-451, AMPK)

We use the following dimensionalization scheme to get the dimensionless key control parameters (Kim et al., 2011a).

(A2) $$\begin{array}{c} \tau =\mu_{1}t,M=\frac{m}{m^{*}},A=\frac{a}{a^{*}},G=\frac{g}{m^{*}},l_{g}=\frac{\Lambda_{g}}{\mu_{1}},\;\\ S=\frac{s}{\mu_{2}a^{*}},l_{1}=\frac{\Lambda_{1}\Lambda_{2}^{2}}{\mu_{1}m^{*}},l_{2}=\Lambda_{2},\\ l_{3}=\frac{\Lambda_{3}\Lambda_{4}^{2}}{\mu_{2}a^{*}},l_{4}=\Lambda_{4},\alpha =\Lambda_{5}{\left(a^{*}\right)}^{2},\;\\ \beta =\Lambda_{6}{\left(m^{*}\right)}^{2},\epsilon =\frac{\mu_{1}}{\mu_{2}}, \end{array}$$

and the dimensionless form of core control system

(A3) $$\begin{array}{ll} \frac{dM}{d\tau } & =l_{g}G+\frac{l_{1}}{l_{2}^{2}+\alpha A^{2}}-M, \end{array}$$

(A4) $$\begin{array}{ll} \epsilon \frac{dA}{d\tau } & =S+\frac{l_{3}}{l_{4}^{2}+\beta M^{2}}-A. \end{array}$$
